# Evaluating Antibody Pharmacokinetics as Prerequisite for Determining True Efficacy as Shown by Dual Targeting of PD-1 and CD96

**DOI:** 10.3390/biomedicines10092146

**Published:** 2022-09-01

**Authors:** Christina Boch, Markus Reschke, Frederik Igney, Peter Maier, Philipp Müller, Sarah Danklmaier, Krishna Das, Tamara Hofer, Guido Wollmann, Wolfgang Rist

**Affiliations:** 1 Boehringer Ingelheim Pharma GmbH & Co. KG, 88400 Biberach an der Riss, Germany; 2 Boehringer Ingelheim RCV GmbH & Co. KG, 1120 Vienna, Austria; 3 Institute of Virology, Medical University Innsbruck, 6020 Innsbruck, Austria; 4 Christian Doppler Laboratory for Viral Immunotherapy of Cancer, 6020 Innsbruck, Austria; 5 ViraTherapeutics GmbH, 6063 Rum, Austria

**Keywords:** CD96, PD-1, anti-drug antibodies, ligand-binding assay, mass spectrometry

## Abstract

One important prerequisite for developing a therapeutic monoclonal antibody is to evaluate its in vivo efficacy. We tested the therapeutic potential of an anti-CD96 antibody alone or in combination with an anti-PD-1 antibody in a mouse colon cancer model. Early anti-PD-1 treatment significantly decreased tumor growth and the combination with anti-CD96 further increased the therapeutic benefit, while anti-CD96 treatment alone had no effect. In late therapeutic settings, the treatment combination resulted in enhanced CD8^+^ T cell infiltration of tumors and an increased CD8/Treg ratio. Measured anti-PD-1 concentrations were as expected in animals treated with anti-PD-1 alone, but lower at later time points in animals receiving combination treatment. Moreover, anti-CD96 concentrations dropped dramatically after 10 days and were undetectable thereafter in most animals due to the occurrence of anti-drug antibodies that were increasing antibody clearance. Comparison of the anti-PD-1 concentrations with tumor growth showed that higher antibody concentrations in plasma correlated with better therapeutic efficacy. The therapeutic effect of anti-CD96 treatment could not be evaluated, because plasma concentrations were too low. Our findings strongly support the notion of measuring both plasma concentration and anti-drug antibody formation throughout in vivo studies, in order to interpret pharmacodynamic data correctly.

## 1. Introduction

According to the theory of “immunosurveillance”, arising cancer cells are initially recognized by the immune system and—in many cases—also eradicated. CD8^+^ T cells and natural killer (NK) cells play an important role in this process because they can identify and kill cancer cells. Hereby, NK cells recognize receptors on the surface of cancer cells that are expressed in response to aberrant cellular behavior, e.g., uncontrolled proliferation. CD8^+^ T cells scan antigens that are presented on the surface of cancer cells (so-called tumor antigens) and identify them as abnormal. Upon binding, both NK cells and CD8^+^ T cells are able to induce apoptosis in the cancer cells unless they are inhibited by different mechanisms [1,2,3]. In particular, as cancer progresses, cancer cells acquire mechanisms that enable them to evade such immune responses, and eventually succeed in hiding from the immune system. This process has been studied extensively, and has led to the identification of many immune-inhibiting mechanisms initiated by the tumor itself or its microenvironment. As an example, distinct cell surface receptors play an essential role in the activation or inhibition of immune cells. One prominent example is the cell surface receptor programmed cell death protein ligand—1 (PD-L1), which has been found on the cell surface of many tumor types. It binds to PD-1 that is expressed, e.g., on CD8^+^ T cells, where it induces a state of functional unresponsiveness apoptosis, thus causing immune evasion of the tumor [4,5].

Therapeutic antibodies targeting such cell surface receptors (e.g., PD-1, CTLA4) have shown remarkable success in the treatment of some malignancies, and have proven that the blockage of so-called immune checkpoints is feasible for cancer therapy [6,7,8]. Notably, PD-1-targeting therapeutic antibodies have become a key player in cancer immunotherapies.

Combinations of existing checkpoint inhibitors have the potential to yield increased anti-tumor immunity [8,9,10,11,12,13], but these also bear the risk of increasing autoimmune reactions [13,14]. In addition, it is being tested if novel checkpoint inhibitors or combinations thereof may result in similar or better response rates while having fewer side effects (reviewed in [15]). A potentially important signaling pathway that is involved in regulating anti-tumor immunity is the T cell immunoreceptor with Ig and ITIM domains (TIGIT)-CD96-CD226 axis. Both T cells and NK cells express TIGIT, CD96 and CD226. They bind to the same ligands, CD155 and CD112, which are overexpressed by tumor cells and associated myeloid and dendritic cells. While the binding of CD155 or CD112 to CD226 activates T and NK cells, their interactions with CD96 and TIGIT result in inhibitory signaling [16]. Therefore, preclinical studies employed inhibitory anti-CD96 monoclonal antibodies (mAbs) to study their potential to induce anti-tumor immunity. It has been shown that a co-blockade of CD96 and PD-1 inhibited tumor growth and lung metastases, and increased local NK-cell infiltration and interferon-γ (IFN-γ) synthesis in several mouse tumor models [17].

Usually, the therapeutic concept of such antibodies is proven in animal models. Since many human-specific mAbs are not species cross-reactive to the orthologous protein, surrogate antibodies are used. These antibodies are often not derived from the same host species as the preclinical model, e.g., rat antibodies are commonly used in mouse models. Prior to in vivo experiments, the affinity and neutralizing capabilities of the antibodies are determined in vitro by employing biochemical binding assays or functional cellular assays. However, it is critical to be aware that these mAbs have immunogenic potential in animal experiments leading to anti-drug antibody (ADA) formation, which can neutralize the mAb (thereby inhibiting its therapeutic function) and lead to immunotoxicity or induce mAb clearance [18]. ADA development is influenced by many factors including not only the protein sequence [19], but also post-translational modifications (in particular glycosylation) [20], formulation [21,22], route of administration [23] and many more (reviewed in [24]). Even humanized mAbs that are optimized for clinical administration induce ADAs in some patients (reviewed in [25]). Despite this, exposure measurements (i.e., quantification in plasma or other tissue specimens) of therapeutic mAbs are often omitted in proof-of-principle studies [17,26,27], although it is important to prove that the antibody has been applied correctly and is available throughout the entire study in order to exert its function.

We carried out an in vivo tumor therapy study in an MC-38 mouse colon cancer model using mAbs to target PD-1 and CD96. We show that anti-PD-1 significantly decreased tumor growth, and that anti-CD96 further increased the therapeutic benefit; meanwhile, anti-CD96 alone had no effect. This anti-PD-1/anti-CD96 combination showed enhanced CD8^+^ T cell infiltration into the tumor. Using a combination of ligand binding assays and mass spectrometry (MS) measurements—with only ten microliters of plasma—we could show that anti-CD96 concentrations were substantially decreased in all animals, and that ADAs were responsible for increased clearance. We show that without such measurements, pharmacodynamic data cannot be properly interpreted, and could lead to an underestimation of the therapeutic efficacy.

## 2. Materials and Methods

### 2.1. Mice

Mice used in these studies were 8–10 week-old C57/BL6NTac mice purchased from Taconic Denmark. After arrival at the animal facility, these mice were allowed to adjust to ambient conditions for at least 5 days before they were used for experiments. They were housed in Macrolon^®^ type III cages in groups of 10 under standardized conditions at 21.5 ± 1.5 °C and 55 ± 10% humidity. Standardized irradiated diet (PROVIMI KLIBA) and autoclaved tap water were provided ad libitum. All animal care and experimental procedures were approved by the local authorities and were in compliance with the local and European Animal Welfare Acts.

### 2.2. Pharmacokinetic Study

Doses of 10 mg/kg of mAbs anti-CD96 (Clone 3.3, Biolegend, San Diego, CA, USA, Cat. No. 131704) or anti-PD-1 (Clone RMP1-14, BioXCell, Lebanon, NH, USA, Cat. No. BE0146) were applied intraperitoneally into C57/BL6NTac mice. Blood samples were drawn before application and after 0.5, 1, 2, 4, 8, 24, 72, 144, 216, 240 and 336 h for anti-PD-1, and after 1, 2, 4, 8, 24, 72, 144, 168 and 216 h for anti-CD96. Pharmacokinetic (PK) parameters were calculated by means of non-compartmental analysis with ToxKin 3.3 software. The expected concentration-time profiles of anti-PD-1 and anti-CD96 mAbs in the tumor therapy study were simulated using Phoenix WinNonLin 6.4.

### 2.3. Tumor Therapy Study

**Figure 1:**
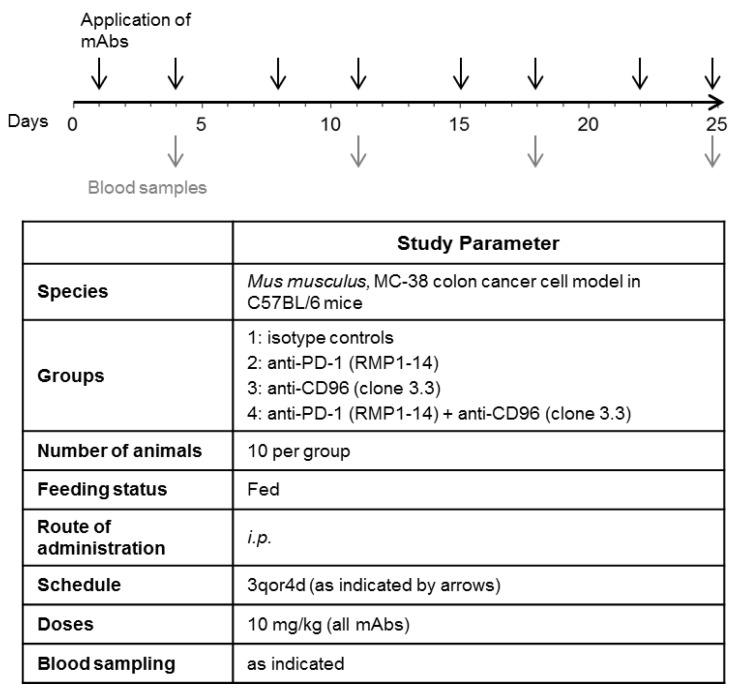
Experimental plan for testing the effect of anti-PD-1 and anti-CD96 antibodies on tumor growth in a mouse colon cancer model. C57/BL6 mice were transplanted with MC-38 colon carcinoma cells on day 0. Treatment with the antibodies was initiated on day 3, post transplantation. Black arrows indicate i.p. application of mAbs, each at 10 mg/kg. Gray arrows indicate drawing of blood samples.

In order to establish the MC-38 tumor model, C57BL/6NTac mice were transplanted with 1 × 10^5^ of MC-38 colon carcinoma cells subcutaneously into the right flank of the mice at day 0. The animals were dispatched randomly by a computer program into the different groups. Application of mAbs was initiated on day 3 after tumor grafting (see Figure 1). The following antibodies were used: anti-CD96 (BioLegend, San Diego, CA, USA, Cat. No. 131704), anti-PD-1 (BioXCell, Lebanon, NH, USA, Cat. No. BE0146) and isotype control antibodies (Rat IgG2a, BioXCell, Lebanon, NH, USA, Cat. No. BE0089 and Rat IgG1 BioXCell, Lebanon, NH, USA, Cat. No. BE0088). The antibodies were diluted in PBS and injected intraperitoneally with a volume of 10 mL/kg per mouse, twice weekly.

### 2.4. Quantification of mAbs by ELISA

Nunc MaxiSorp ELISA plates were coated overnight at 4 °C with 100 µL per well of 500 ng/mL mouse anti-rat IgG1 (USBiological Life Sciences, Swampscott, MA, USA, Cat. No. I1904-75M) for the detection of anti-CD96 in the tumor therapy study, or 200 ng/mL recombinant mouse CD96 (R&D Systems, Cat. No. 5690-D-050) for the detection of anti-CD96 in the PK study, or 40 ng/mL mouse anti-rat IgG2a (Sigma Aldrich Corp., St. Louis, MO, USA, Cat. No. R0761) for the detection of anti-PD-1 diluted in PBS. Blocking was performed by adding 200 µL of blocking buffer (PBS containing 1% Casein) for at least 1 h at 4 °C. After washing the plates 4 times with 450 µL washing solution (0.05% Tween 20), 100 µL of the samples that were diluted in sample buffer (PBS with 0.05% Tween 20, 0.1% Casein) were added. The dilution of the samples was chosen according to their expected concentration. A calibration curve of the mAb in triplicates in the same buffer with the appropriate matrix concentration was included in every plate. The sealed plates were incubated for 1 h at room temperature with shaking. Plates were washed again 4 times with 450 µL of washing solution and 100 µL of 50 ng/mL horseradish peroxidase (HRP)-conjugated donkey anti-rat IgG (Jackson Immuno Research Cat. No. 712-035-153) for anti-CD96, or 40 ng/mL HRP-conjugated goat anti-rat IgG2a (Acris Antibodies Cat. No. AP05379HR-N) for anti-PD-1 diluted in sample buffer was added. The sealed plates were incubated for 1 h at room temperature (shaking) followed by another wash step (4 times with 450 µL of washing solution). Afterwards, 100 µL of TMB solution (Sigma-Aldrich Cat. No.T0440) was added, then incubated for 20–30 min in the dark and stopped by the addition of 100 µL of 1M HCl. The absorption at 450 nm was measured using a SpectraMax 340PC-384 photometer. The software SoftMaxPro6.5 was used for analysis; the calibration curves were fitted using a 4-parameter logistic function.

### 2.5. MS-Based Quantification of Total mAbs

Amounts of 9 µL of denaturing buffer (50 mM Tris/HCl, 8 M Urea, 20 mM TCEP) were added into each well of a 96-well plate. Then, 2 µL of the plasma sample was added. Thereby, the proteins were denatured and reduced. A calibration curve of anti-CD96 and anti-PD-1 in mouse C57/BL6 plasma was included, ranging from 1–5000 nM. An amount of 4 µL of alkylation buffer that contained 50-millimolar iodoacetoamide and 400-nanomolar SILu™MAB heavy standard (Sigma-Aldrich Cat. No. MSQC3) in 50-millimolar TEAB was added to alkylate cysteine side chains. Samples were incubated for 20 min at room temperature in the dark and subsequently treated with 1.3 µL of 3 µg/µL endoproteinase Lys-C (Wako Cat. No. 121-05063) for 3 h at 37 °C. Thereafter, 57 µL of 0.07 µg/µL trypsin (Promega Cat. No. V5280) in 50-millimolar TEAB were added. The samples were incubated over night at 37 °C. Subsequently, 5 µL of 3% formic acid were added to stop the reaction, and the samples were analyzed using liquid chromatography with tandem mass spectrometry (LC-MS/MS) on a QTRAP 6500+ system (AB Sciex, Toronto, ON, Canada), under the conditions shown in Table S1. Analyst 1.6.3 software was employed for quantifying anti-CD96 and anti-PD-1. The calibration curves were fitted with a linear regression and 1/x^2^ weighting.

### 2.6. ADA Assay

Anti-CD96 and anti-PD-1 mAbs were biotinylated with EZ-Link™ Sulfo-NHS-LC-Biotin (Thermo Fisher Scientific Cat. No. 21335) and sulfo-tagged with MSD GOLD SULFO-TAG NHS-Ester (Meso Scale Discovery Cat. No. R91AO-1), according to the manufacturer’s protocol. For detecting ADAs against anti-CD96, a master mix was prepared by mixing biotinylated and sulfo-tagged anti-CD96 in antibody buffer (PBS with 0.05% Tween 20, 0.1% Casein) at a final concentration of 2.5 µg/mL. For detecting ADAs against anti-PD-1, biotinylated and sulfo-tagged anti-PD-1 were mixed in antibody buffer to a final concentration of 1 µg/mL. The optimal concentrations of sulfo-tagged and biotinylated antibodies had been determined in a preceding experiment. Plasma samples were diluted in MSD sample buffer (PBS with 0.05% Tween 20, 1% BSA) to ratios of 1:12.5 for detecting anti-PD-1 ADAs, or to 1:20 for detecting anti-CD96 ADAs. Positive controls were prepared at different concentrations at the same dilutions. A reaction mixture was prepared by pipetting 50 µL of antibody master mix and 25 µL of diluted samples in a 96-well plate and incubated for 2 h at room temperature on a shaker. An MSD STREPTAVIDIN GOLD^TM^ plate (Meso Scale Discovery, Cat. No. L15SA) was blocked by the addition of 150µL of MSD blocking buffer (PBS with 3% BSA) per well and incubated at room temperature for at least 30 min. Subsequently, the blocked MSD plate was washed three times with 450 µL of washing buffer (0.05% Tween 20), and 50 µL of the pre-incubated reaction mixture was added to each well followed by an incubation for 2 h, with shaking at room temperature. Thereafter, the MSD plate was washed again three times, and 150 µL of Read Buffer T 2x (Meso Scale Discovery Cat. No. R92TC-3) was added. The plates were read out on an MSD Sector Imager 6000 using the Sector Image Software.

### 2.7. Analysis of Tumor Tissue and Infiltrating Lymphocytes

Mice with established and sizeable MC-38 tumors (14 days post grafting) were grouped into equal tumor distributions, after which antibody treatment was initiated. Mice were treated intraperitoneally three times every three to four days with anti-CD96 (Ultra-LEAF^TM^ Purified anti-mouse CD96 (TACTILE) Clone: 3.3, Biolegend), anti-PD-1 (Anti-mouse PD1-rat IgG2a Clone: RMP1-14, BioXCell), or isotype control (GoInVivo^TM^ Purified Rat IgG2a,k Isotype Ctrl Clone:RTK2758 and GoInVivo^TM^ Purified Rat IgG2a,k Isotype Ctrl Clone:RTK2758) antibodies with a concentration of 10 mL/kg per mouse. Eleven days after mAb treatment, tumors were harvested for tumor infiltrating lymphocyte (TILs) analysis. Tumors were digested using the mouse tumor dissociation kit (Miltenyi Biotec, Bergisch Gladbach, Germany) in combination with the gentleMACS™ dissociator (Miltenyi Biotec), following manufacturers’ instructions. The single-cell suspension was filtered through a 70-micrometer cell strainer; erythrocytes were lysed using Pharm Lyse^TM^ Lysing buffer (BD Bioscience, San Jose, CA, USA), and FC block (Purified Rat Anti-Mouse CD16/CD32, BD Biosciences) was performed for all samples. The following mouse antibodies were used for surface and intracellular staining: CD45.2 (clone 104, Biolegend), CD8a (clone 53-6.7, Biolegend), CD3 (clone 17A2, Biolegend), CD4 (clone GK1.5, Biolegend), CD25 (clone PC61, Biolegend), NK1.1 (clone PK136, Biolegend), CD96 (clone 3.3, Biolegend), CD279 (PD-1) (clone 29F.1A12, Biolegend) and Foxp3 (clone NRRF-30, eBioscience). For intracellular staining, cells were fixed and permeabilized using the transcription factor buffer set (BD Bioscience). In order to exclude dead cells, LIVE/DEAD™ Fixable Near-IR Dead Cell Stain Kit (Thermo Fischer Scientific) was used. Samples were acquired using FACS Canto II (BD Biosciences), and the data were analyzed with FlowJo software version 10.5.3 (FlowJo LLC, Ashland, OR, USA).

For transcriptome analysis, MC-38 tumors were homogenized with the SpeedMill PLUS (Analytik Jena, Jena, Germany), and RNA was extracted using phenol: chloroform:isoamyl alcohol (25:24:1) (Sigma-Aldrich, USA) and MagMAX-96 Total RNA Isolation Kit (Thermo Fisher Scientific, Waltham, MA, USA), according to the manufacturer’s instructions. The mRNA count was analyzed for differential expression by means of the nCounter PanCancer Immune Profiling Panel and the nCounter FLEX Analysis System (NanoString Technologies, Seattle, WA, USA).

### 2.8. Statistical Analysis

The statistical evaluation was performed for the parameter tumor volume on day 15 of the study. Due to the observed variations, nonparametric methods were applied. For descriptive considerations, the median was calculated. For a quick overview of possible treatment effects, the median of the tumor volume of each treatment group T was referred to the median of the control C as follows:

Tumor growth inhibition (TGI) from day 1 until day 15

TGI = 100 × [(C_15_−C_1_)−(T_15_−T_1_)]/(C_15_−C_1_) (1)

where C_1_ and T_1_ are median tumor volumes in the control and treatment groups at day 1, respectively, while C_15_ and T_15_ represent median tumor volumes in the control and treatment groups at day 15, respectively.

One-sided nonparametric Mann–Whitney–Wilcoxon U-tests were applied to compare each treatment group with the control, looking for a reduction in tumor volume as an effect. In this study, the U-test compares the ranking of the individual tumors of two groups according to absolute volume on a particular day (pairwise comparisons between groups).

The *p*-values were adjusted for multiple comparisons according to Bonferroni–Holm within each subtopic (comparisons of therapies versus control, comparisons of mono therapies versus combination therapy). For TILs analysis, one-way ANOVA was applied followed by Tukey’s multiple comparison test. For all tests, the level of significance was fixed at α = 5%. An (adjusted) *p*-value of less than 0.05 was considered to show a statistically significant difference between the groups.

## 3. Results

According to Blake et al., treatment with anti-CD96 protected mice against experimental lung metastases in three different models; this effect was more pronounced when combined with anti-PD-1 [17]. In order to test whether anti-CD96 (alone or in combination) can also reduce tumor growth in a mouse colon cancer model, we treated C57BL/6 mice bearing a syngeneic subcutaneous MC-38 tumor (MC-38 mice) in a tumor therapy study twice a week as indicated (Figure 1), with anti-PD-1, anti-CD96, a combination of both antibodies, or isotype controls. We used the same mAbs, which bind and functionally inhibit the target receptors as previously shown by cellular assays, and in vitro as well as in vivo studies [17,28,29,30,31]. In order to corroborate previous findings that showed CD96-mediated immune suppression in the MC-38 tumor model, we first confirmed via flow cytometry the expressions of CD96 on both tumor infiltrating T cells and NK cells (Figure S1A), as well as the expression of the CD96 main ligand, CD155, on non-leukocytic cells (Figure S1B). We also assessed the quantitative expression levels of CD155 and PD-L1 using NanoString transcriptome analysis. Untreated MC-38 tumors showed comparable mRNA levels for both the CD96 ligand CD155 and the PD-1 ligand PD-L1 (Figure S1C).

**Figure 2:**
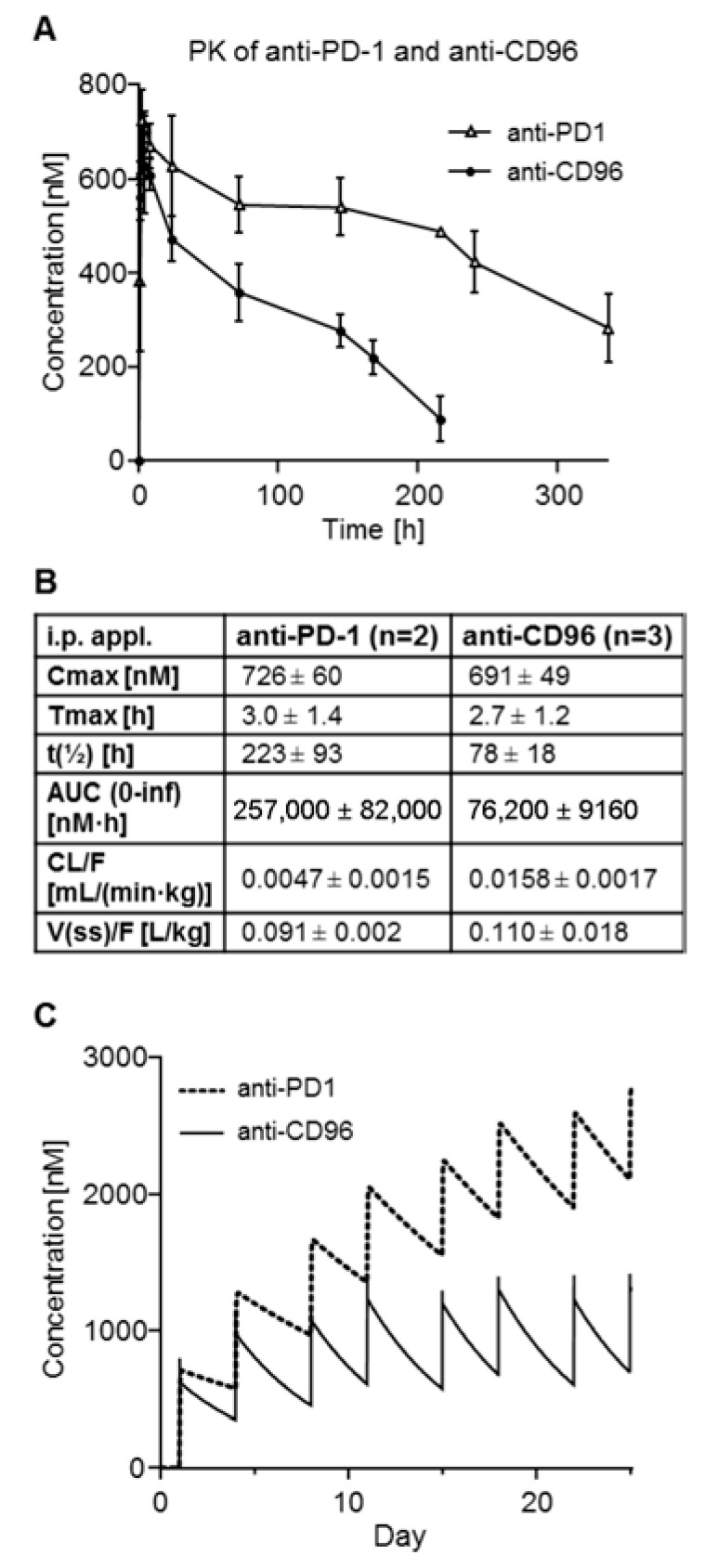
PK studies of anti-PD-1 and anti-CD96 in mice after single i.p. application. (**A**) Mean plasma concentration–time profiles of anti-PD-1 and anti-CD96 as determined by ELISA; (**B**) mean PK parameters calculated by non-compartmental analysis; (**C**) simulated concentration–time profiles of anti-PD-1 and anti-CD96 for the tumor therapy study.

In order to predict the plasma concentrations of anti-CD96 and anti-PD-1, PK studies in C57/BL6 mice were carried out. The concentration–time profiles are shown in Figure 2A, and the calculated PK parameters are shown in Figure 2B. Although we did not expect major differences between these two antibodies regarding pharmacokinetics, the area under the curve (AUC) and the terminal half-life (t_1/2_) of anti-PD-1 mAb (rat IgG2a isotype) was about three times higher compared to those of anti-CD96 mAb (rat IgG1 isotype). The simulated concentration–time profiles of both mAbs for the tumor therapy study are shown in Figure 2C. Despite of the PK differences, the application scheme should result in high mAb concentrations throughout the experiment, thus allowing the antibodies to exert their anti-tumor effects.

**Figure 3:**
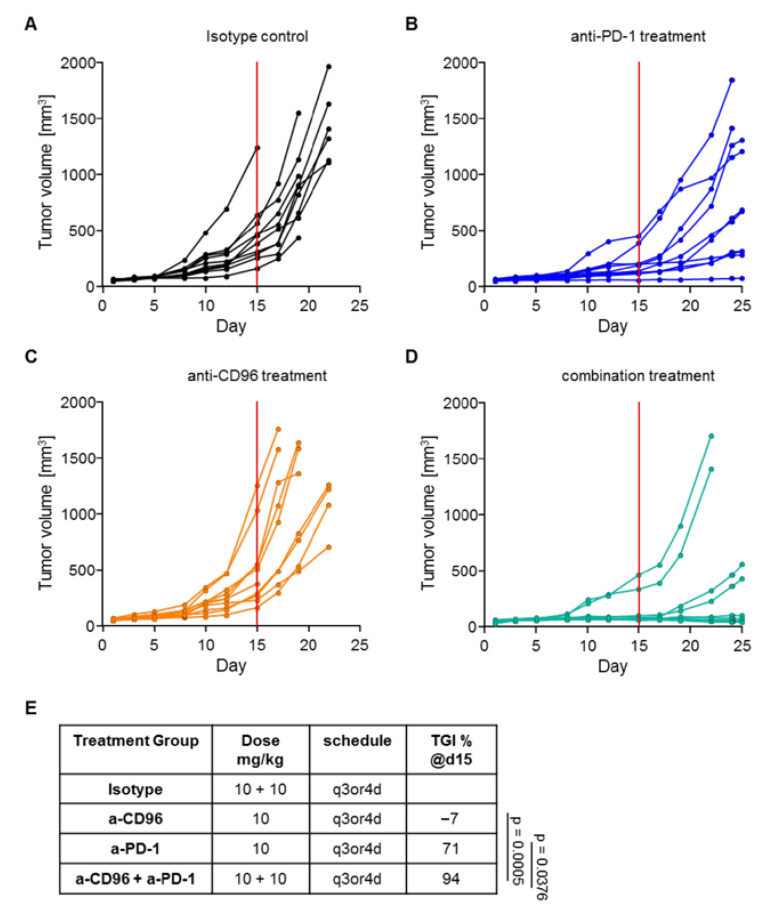
Tumor growth in mice treated with mAbs. Tumor volumes are shown for mice treated with (**A**) isotype control antibodies; (**B**) anti-PD-1; (**C**) anti-CD96; and (**D**) the combination of anti-PD-1 and anti-CD96. (**E**) Tumor growth inhibition (TGI) measured on day 15 (red line in graphs A-D) of the experiment for all treatment groups.

As shown in Figure 3, early initiation of anti-PD-1 treatment in MC-38 mice resulted in a significant reduction in tumor growth compared to the control group, while anti-CD96 alone did not have any effect. However, mice treated with both antibodies showed significantly reduced tumor growth compared to the isotype control or to the anti-CD96 or anti-PD-1 treatment groups. Plasma samples of five animals per group were retained for mAb quantification. Without exposure data, one could argue that anti-CD96 treatment alone had no therapeutic effect, while it was beneficial in combination with anti-PD-1.

**Figure 4:**
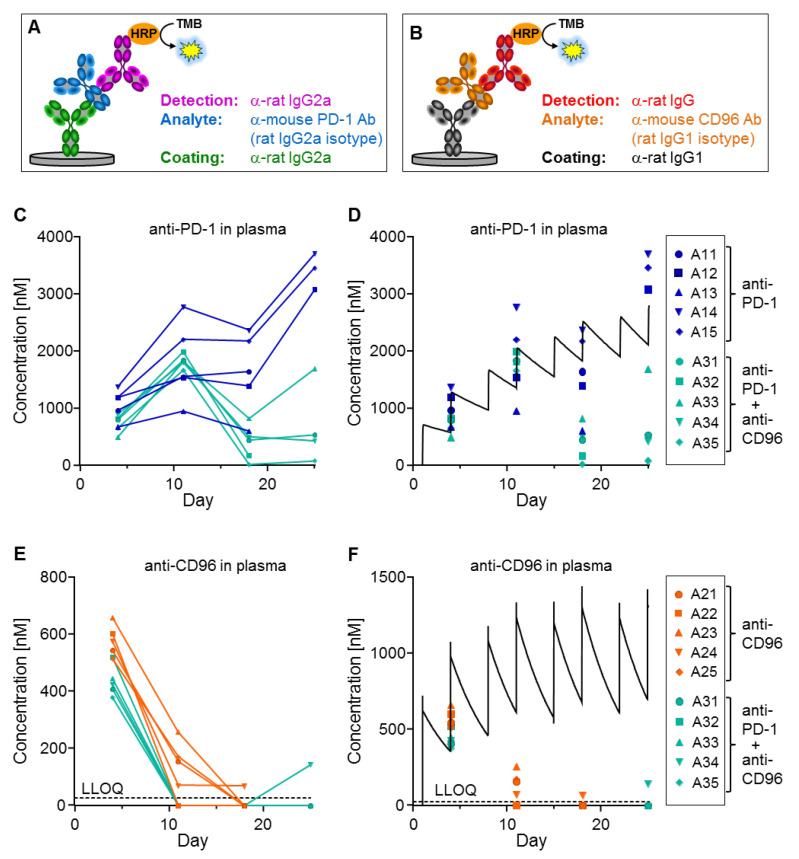
Exposure measurement of tumor therapy study. (**A**,**B**) ELISA setup against anti-PD-1 and anti-CD96. (**C**,**E**) Plasma concentrations of anti-PD-1 and anti-CD96 were determined for 5 animals per group and plotted against time. Samples below LLOQ are plotted on the *x*-axis. (**D**,**F**) Simulated concentration–time curves (black lines, see Figure 2C) are compared with the measured data for anti-PD-1 and anti-CD96, respectively. Treatment groups are shown on the right. Plasma samples for animals 11, 13, 21–25 and 32 at day 25 are missing because these animals were sacrificed due to high tumor load.

Using the ELISA assays described in Figure 4A,B, plasma levels of the mAbs in the tumor therapy study were measured using only two microliters of plasma. Anti-CD96 reached the expected concentrations at day 5, but surprisingly, levels dropped substantially after the third application. Specifically, the anti-CD96 concentrations at day 11 were below 300 nM for animals treated with anti-CD96 alone, or even below the lower limit of quantification (LLOQ) of 26.7 nM for animals receiving both antibodies (Figure 4E,F). The anti-PD-1 levels were influenced more differentially; while animals receiving the single antibody reached the expected concentrations (except for animal 13), mice that were treated with both antibodies had reduced concentrations at days 18 and 25 (Figure 4C,D).

**Figure 5:**
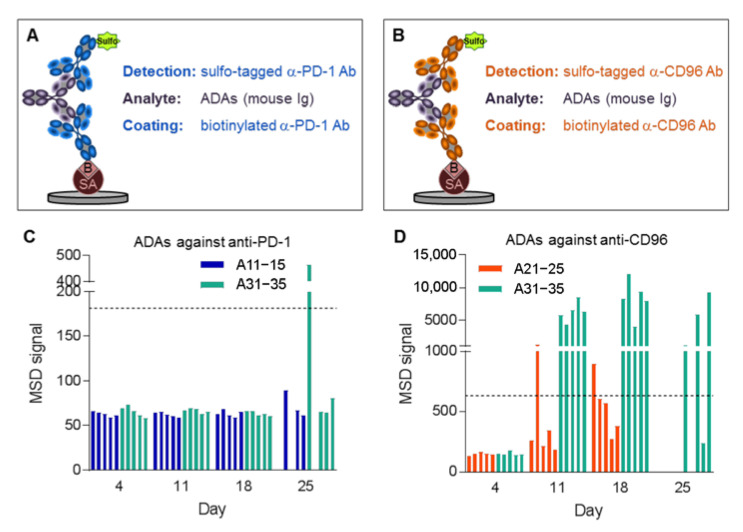
ADAs against anti-CD96 and anti-PD-1. ADA assays on the MSD platform against (**A**) anti-PD-1 and (**B**) anti-CD96 using streptavidin (SA)-coated plates. MSD signal for measurement of ADAs against (**C**) anti-PD-1 and (**D**) anti-CD96. Horizontal lines indicate MSD signals three times above background that are considered positive for ADAs. Plasma samples for animals 11, 13, 21–25 and 32 at day 25 are missing because these animals were sacrificed due to high tumor load.

We hypothesized that ADAs were responsible for the observed low mAb concentrations. Therefore, bridging assays on the MSD platform were developed for detecting ADAs against anti-PD-1 and anti-CD96 (Figure 5A,B), respectively. For this purpose, the mAbs were labeled with either biotin or sulfo-tag, leading to a positive MSD signal when ADAs are present in a sample. This assay enabled us to measure ADAs in the plasma samples of the MC-38 mice, using only two microliters of plasma. Plasma samples were considered positive for ADAs if their signal was increased by more than three times above background. It is important to note that the sensitivity of the ADA assays may be too low to detect small amounts of high-affinity ADAs, or even higher amounts of low-affinity ADAs present at the beginning of the immunogenic reaction. For background measurement, plasma samples of untreated mice were used. Figure 5D shows that many animals had ADAs against anti-CD96 beginning at day 11, especially those treated with both mAbs. The intensities of the MSD signals were only moderately increased in animals receiving anti-CD96 alone (A21–25), but excessively high in animals that were additionally treated with anti-PD-1 (A31–35). ADAs against anti-PD-1 (Figure 5C) were only detected in one animal (A31) receiving the antibody combination at day 25. Taken together, these data suggest that ADAs were responsible for the detection of low concentrations of the mAbs.

**Figure 6:**
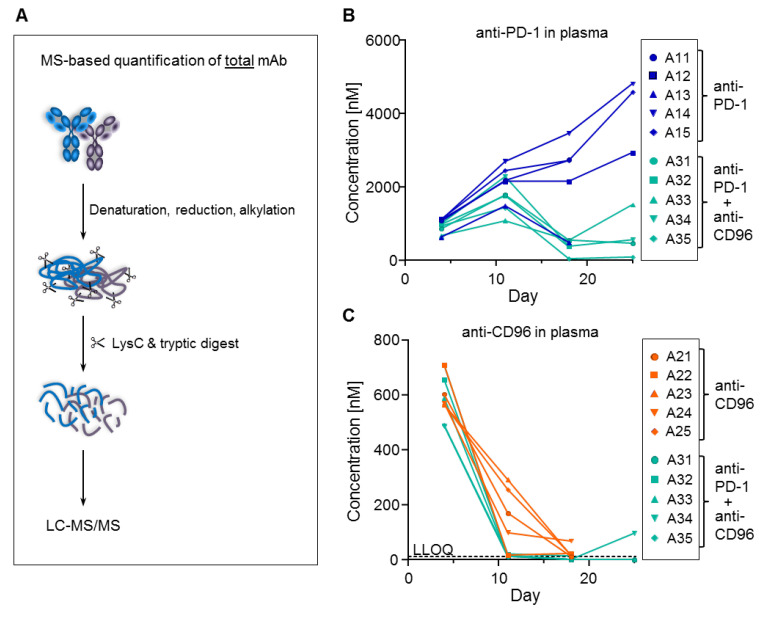
Total concentrations of anti-PD-1 and anti-CD96 measured by MS. (**A**) MS-based workflow for total mAb quantification. Total plasma concentrations of (**B**) anti-PD-1 and (**C**) anti-CD96 (compare with ELISA data from Figure 4). Samples below LLOQ are plotted on the *x*-axis. Plasma samples for animals 11, 13, 21–25 and 32 at day 25 are missing because these animals were sacrificed due to high tumor load.

In terms of mAb measurement, the ADAs can have two different effects. First, they might interfere with the ELISA assay by binding to the same epitope as either the coating or the detection antibodies, thereby causing analytical artifacts. Second, they might increase mAb clearance by inducing endocytosis of the ADA/mAb complex in phagocytic cells such as macrophages. The best way to distinguish between the two possibilities is to measure the total concentration of the mAbs (i.e., free and bound fractions). For this purpose, a method that is independent of any antibody-based enrichment is required since any binding site of the mAb may be occupied by ADAs. Therefore, we denatured and digested a two-microliter plasma sample with trypsin and quantified a tryptic surrogate peptide of the mAbs by LC-MS/MS, achieving an LLOQ of 10 nM (Figure 6A). We selected tryptic peptides that are specific and selective for anti-PD-1 and anti-CD96; Figure 6B,C, respectively, show that these total mAb concentrations measured by MS were almost identical to those determined by ELISA (compare with Figure 4C,E). These data prove that the ADAs did not interfere with the ELISA assay, and hence were responsible for increased clearance. It is noteworthy that only ten microliters of mouse plasma were used to perform all these bioanalytical experiments, i.e., ELISA, ADA and MS measurements.

**Figure 7:**
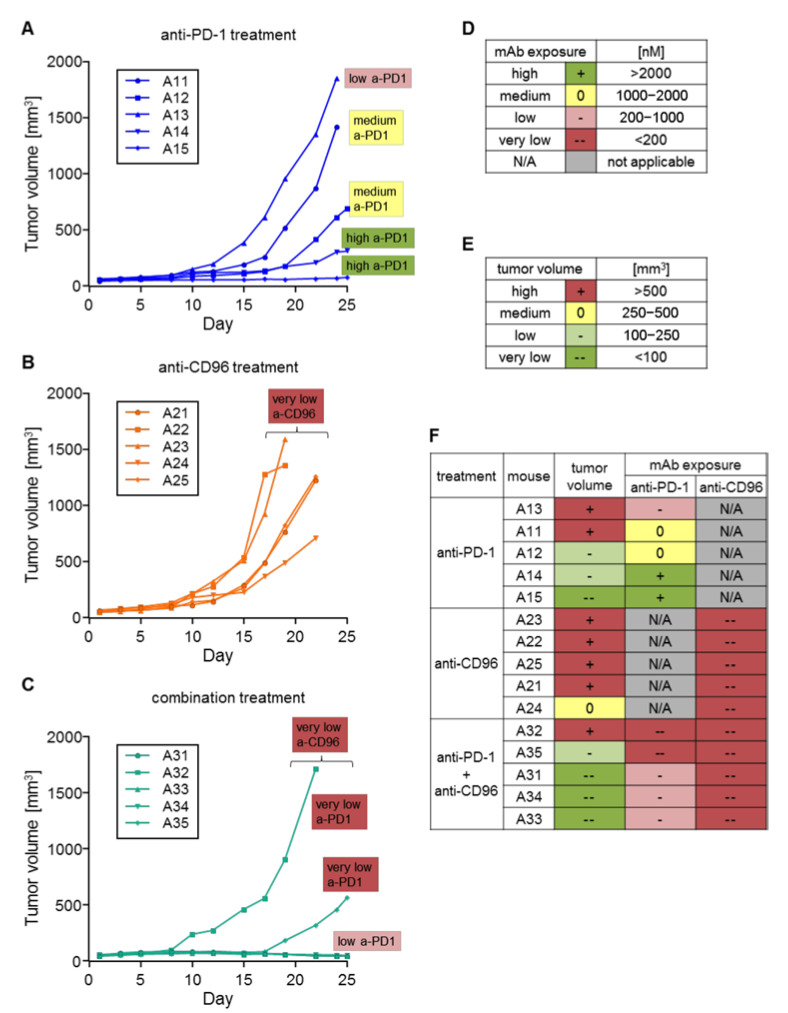
Comparison of tumor growth with mAb exposure. An increase in tumor volume is shown for mice treated with (**A**) anti-PD-1; (**B**) anti-CD96; and (**C**) the combination of anti-PD-1 and anti-CD96. (**D**) Categorizations of antibody exposure; (**E**) categorizations of tumor size; (**F**) comparison of antibody exposure (measured at day 18) with tumor size (measured at day 19). Mice are sorted by treatment groups and tumor sizes at day 19.

Since the mAb concentrations were extremely variable between different treatment groups and also within the same group, we were interested to see the consequences of high or low mAb exposure on tumor growth. Therefore, mAb exposure was divided into four categories according to the concentration at day 18 measured by ELISA: high(+): >2000 nM; medium(0): 1000–2000 nM; low(−): 200–1000 nM; very low(−−): <200 nM (Figure 7D). Similarly, tumor volume at day 19 was divided into four categories: high(+): > 500 mm^3^; medium(0): 250–500 mm^3^; low(−): 100–250 mm^3^; very low(−−): <100 mm^3^ (Figure 7E). Days 18 and 19 were chosen because these are the last time points when data were available for all animals, as some animals were sacrificed thereafter due to having tumor volumes greater than 1500 mm^3^. In the anti-PD-1 group (Figure 7A), the mouse with the lowest mAb concentration (A13) showed the largest tumor. On the other hand, the two animals with high mAb exposure (A14, 15) had the lowest tumor volumes in this group. The mAb exposure of mice treated with anti-CD96 alone was very low for all five mice (A21–25), and tumor volumes were high or medium (Figure 7B). Mice treated with both mAbs (A31–35) also had very low anti-CD96 exposure and on top of that, only very low to low anti-PD-1 exposure. Mice with low anti-PD-1 exposure (A31, 33, 34) had very low tumor sizes, while those with very low anti-PD-1 exposure (A32, 35) had low to high tumor sizes (Figure 7C). Interestingly, whereas low anti-PD-1 exposure in animals treated with anti-PD-1 alone allowed the tumor to grow fast (A13), a similar low exposure in the combination group (A31, 33 and 34) showed regression in tumor growth, although anti-CD96 levels were below LLOQ. It seems that short-term anti-CD96 exposure at the beginning of the experiment was sufficient to have beneficial effects on top of anti-PD-1 treatment, but not alone.

**Figure 8:**
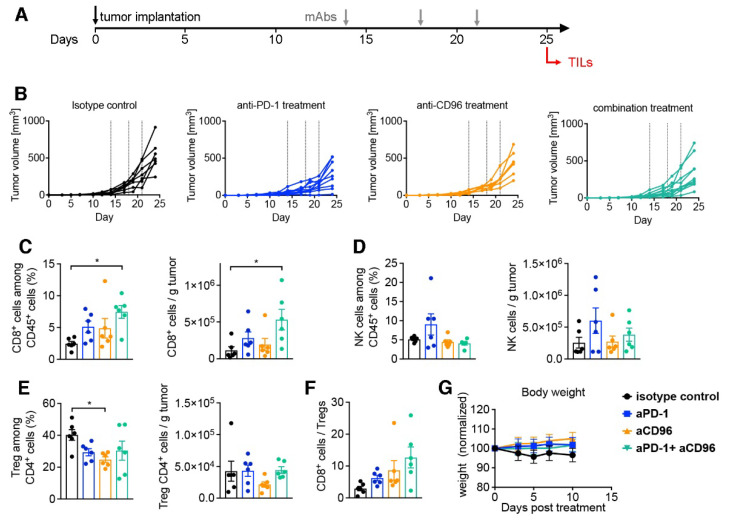
Analysis of tumor infiltrating lymphocytes in mice treated with mAbs. C57BL/6NTac mice were injected s.c. with MC-38 tumors on day 0; mAb treatment commenced at a delayed therapeutic setting 13 days later. Tumors were harvested on day 25 for flow cytometric analysis. (**A**) Schematic of experimental outline; (**B**) tumor volumes are shown for mice under the respective treatment. Dotted lines indicate treatment time points. CD8^+^ cells (**C**), NK cells (**D**) and regulatory CD4^+^ Treg cells (**E**) were quantified in tumor tissues and displayed both as frequency and number; (**F**) ratio of CD8^+^ T cells to CD4^+^ Treg cells; (**G**) weight chart normalized to weight at treatment initiation expressed as mean ± SEM (*n* = 6). Bar graphs in (**C**–**F**) are displayed as mean ± SEM (*n* = 6) with symbols reflecting individual mice. * *p* < 0.05, one-way ANOVA followed by Tukey’s multiple comparison test.

In order to further corroborate the beneficial effect of the anti-CD96/anti-PD-1 combination, we investigated the composition of tumor-infiltrating lymphocytes in MC-38 tumors after 11 days of treatment. This harvest timepoint was chosen as a compromise between detectable mAbs levels and the emergence of ADAs. However, to ensure consistent presence of harvestable tumor material we opted to initiate antibody treatment 14 days after tumor placement, by which time sizeable tumors had formed in every mouse (Figure 8A). Consequently, the therapeutic effect was less pronounced compared to our initial tumor study shown above (Figure 8B). Importantly, we could detect an increase in CD8^+^ T cells in tumors of mice treated with the dual antibody combination compared to anti-PD-1 or anti-CD96 alone, reaching statistically significant levels compared to isotype control (Figure 8C). In contrast, no statistically significant effects were found on NK cells (Figure 8D). Anti-CD96 treatment could also significantly reduce the frequency of regulatory CD4^+^ T cells (Treg) compared to control, although this was less pronounced in the combination with anti-PD-1 (Figure 8E). Finally, although not statistically significant, the effector/regulatory ratio CD8^+^/Treg was favorably shifted by the anti-CD96/anti-PD-1 co-blockade (Figure 8F). Of note, the combination of both antibodies did not result in changes to the animals’ well-being or weights (Figure 8G).

Altogether, these results indicate that the pharmacodynamic effects of anti-CD96 alone could not be determined, and are underestimated since sufficient plasma concentrations were not reached due to ADA formation. Despite short and low exposure, anti-CD96 had a significant and durable effect in combination treatment with anti-PD-1, resulting in increased tumor infiltration of CD8^+^ T cells and an increased CD8/Treg ratio. With sufficient plasma levels, the anti-tumor effect of anti-CD96 alone or in combination may potentially be much better than these experiments suggest.

## 4. Discussion

The CD226-TIGIT-CD96 axis is involved in T cell and NK cell anti-tumor responses and may represent an attractive target for immunotherapy. It has been shown that a co-blockade of anti-PD-1 and anti-CD96 decreases metastases in murine tumor models and results in superior tumor control [17,32,33]. Here, we studied the effect of anti-CD96 alone and in combination with anti-PD-1 on tumor growth in the mouse colon cancer model MC-38. Previous reports have shown this cell line to express the CD96 ligand CD155 in vitro as well as in vivo, with the important finding that its expression is significantly enhanced within the tumor mass compared to non-tumor control tissue [34]. We therefore first confirmed the presence of CD155 in our MC-38 colon cancer mouse model through both cytometric analysis (gating on CD45 negative cells) and RNA expression levels. For the latter, the use of NanoString transcriptome analysis allowed for an amplification-free comparison of counts for both the CD155 and PD-L1 mRNA, which revealed similar expression levels. Accordingly, our data on PD-L1 expression in the MC-38 tumor model are in line with previous reports, including those with immune-histochemical confirmation [35]. We used the same rat-derived mAbs that had been employed by Blake et al. [17]. We observed a reduction in tumor growth for mice treated with anti-PD-1 alone, and a greater effect when anti-CD96 was applied additionally, whereas anti-CD96 alone had no effect. However, the therapeutic effect of anti-CD96 treatment may be underestimated since the concentrations of anti-CD96 in the mice were dramatically lower than expected due to ADA formation.

This research, in addition to other preclinical studies testing (surrogate) mAbs in tumor-bearing animals, are an essential prerequisite for proof-of-concept of therapeutic antibodies. Nevertheless, many in vivo mice studies with mAbs have been conducted with xenogenic rat antibodies [17,33], and the development of efficacy-interfering ADAs is a valid possibility. In these studies, the mAb concentrations in the plasma of experimental animals throughout the experiment are not shown, hence it is unknown whether or not the mAbs were present at a sufficient concentration and thus capable of exerting their potential therapeutic effect. Indeed, mAbs may have an increased clearance because of target-mediated drug disposition (TMDD) or ADA-induced immune complex formation [25].

In order to understand the PK of our mAbs and to predict the concentrations of anti-CD96 and anti-PD-1 in the tumor therapy study, we performed PK studies. Subsequently, we monitored the mAb concentrations during the tumor therapy study using ELISA and MS measurements. For animals treated with anti-PD-1 alone, the measured concentrations corresponded well with the predicted levels, indicating that it is useful to predict mAb concentrations based on preceding PK studies. In all the other animals, however, we observed concentrations that were lower than expected. In particular, anti-CD96 concentrations were substantially lower than predicted in all mice. Thus, the absence of a therapeutic effect in animals treated with anti-CD96 alone does not necessarily indicate that the target is not useful or that the antibody is not potent enough; however, it may just be a consequence of its low systemic availability. This demonstrates that it is essential to quantify mAbs, especially in sub-chronic in vivo studies.

Plasma concentrations of anti-CD96 began to drop at day 11. Therefore, we suspected that ADAs were developed by the animals because endogenous antibodies usually occur, at the earliest, five to six days after antigen encounter, and are favored by multiple antigen occurrences [36]. For this reason, we developed ADA assays in the MSD bridging format. Since the ADA response is polyclonal, the assay is not suitable for absolute quantification. Nevertheless, a high MSD signal is an indication for a strong ADA response, suggesting that medium and large levels of ADAs against anti-CD96 were formed in animals that were treated with anti-CD96 alone and in combination with anti-PD-1, respectively.

ADAs against anti-PD-1 were only detectable in one animal that received the antibody combination (A31). However, since the anti-PD-1 concentrations were very low in most mice of this group, it is very likely that they also developed ADAs against anti-PD-1, which were not detected due to insufficient assay sensitivity and strong sample dilution.

When we determined the mAb concentrations, we initially used ELISA. However, ELISAs may be susceptible to the presence of ADAs [37]. In particular, ADAs could interfere with the binding of the capture or the detection antibody to the therapeutic mAb, thereby decreasing the signal. It is therefore impossible to distinguish whether ADAs interfere with the assay or increase the clearance of the mAb. Although ELISA is still widely considered to be the gold standard for measuring mAbs, this drawback must be taken into account when interpreting ELISA data. Therefore, a different method was required, one that is not influenced by ADAs, in order to measure the mAb concentrations. We used an MS-based approach that included sample denaturation and digestion by trypsin. For the quantification of the mAbs, we selected a tryptic peptide that is uniquely present in the mAb, but not in any endogenous mouse protein, and shows a good response in MS measurements. Similarly with ELISA, we generated a calibration curve by spiking the mAbs into mouse plasma. Strikingly, we measured nearly the same values as with ELISA, proving that ADAs did not interfere with the ELISA but increased the clearance of the mAbs—this is in line with the observation that animals with very low mAb exposure had very high ADA signals. This suggests that the anti-CD96 and anti-PD-1 antibodies induced considerably different amounts of ADAs, indicating that the mAbs triggered very different extents of immunogenicity in the mouse colon cancer model. The PK study was not capable of predicting ADA development, probably because the antibodies were injected only once. The combination of both mAbs induced a stronger ADA response than the single treatments, leading to lower antibody levels for both antibodies. As both antibodies are of rat origin, anti-CD96 may have triggered an immunogenic reaction that cross-reacted with the anti-PD-1 antibody, so that the immune response became further amplified. Alternatively, the potential synergistic immunostimulatory function of anti-CD96 and anti-PD-1 may have boosted the ADA response.

These results suggest that ADA assays should be employed in preclinical in vivo studies using therapeutic mAbs. Even if ADAs do not increase clearance, they might neutralize the mAb or induce unwanted immune reactions. During clinical studies with mAbs, ADA assays are a key aspect because immune reactions against mAbs can influence the effectiveness of the drug, or even lead to severe adverse events [38]. Albeit safety concerns are not as important in preclinical studies, ADA assays are essential to correctly interpret the results.

As the mAb concentration in this study was dramatically different between and within groups, the question arose if mAb exposure correlated with tumor growth. Indeed, a therapeutic effect of anti-PD-1 alone was only observable in animals with high or medium levels of mAbs. As anti-CD96 levels were very low or absent in all animals receiving anti-CD96 alone or in combination with anti-PD-1, a therapeutic effect was very unlikely. This observation underlines the importance of determining mAb concentrations throughout an animal study. Additionally, it corroborates the hypothesis that the effect of mAbs cannot be compared with each other unless their exposure is determined. Animals receiving both mAbs had very low anti-CD96 concentrations, and anti-PD-1 levels were substantially lower than in animals treated with anti-PD-1 alone. Nevertheless, a reduction in tumor growth was observed in four of five animals. If higher plasma concentrations of anti-CD96 would have been reached, its potential to reduce tumor growth might have been much better. Hence, its efficacy in our tumor therapy study may have been underestimated. On the other hand, in a late treatment onset study that began 14 days post tumor placement, we detected increased CD8^+^ T cell infiltration in tumors in the combination treatment group. This is in line with previous reports on the CT26 tumor model [33]. CD96 has been shown to affect NK function in tumors [39]. In our TILs analysis we could not detect an effect of the CD96 blockade on frequency or absolute count of NK cells, although their functional assessment was not performed. Little is known about the role of a CD96 blockade on the inhibitory immune cell fraction constituted by Tregs [39], with one study showing no effect of anti-CD96 on CD4^+^ Tregs [33]. Our analysis revealed a slight reduction in Treg frequency in MC-38 tumors in the anti-CD96 treatment group. The increased CD8^+^ and reduced Treg frequencies resulted in a positive shift of the CD8^+^/Treg ratio, which importantly has also been described for this tumor model in CD96/PD-1 dual knockout mice [40]. Together, we were able to corroborate previous findings of PD-1 and CD96 co-inhibition on the TILs composition in murine colon tumor models.

Interestingly, Blake et al., amongst others, studied the effect of anti-CD96 and anti-PD1 in a fibrosarcoma mouse model over a period of several weeks and observed only a modest effect [17]. In contrast, Mittal et al. reported reduced tumor growth from an anti-PD-1 and anti-CD96 co-blockade in the murine colon tumor model CT26 [33]. One possible explanation could be ADA formation and increased (rat) mAb clearance, which is also in line with the fact that the mAbs were more effective in different metastases models that involved shorter treatment periods [17].

Last but not least, we did not observe apparent signs of clinical toxicity, which can be a concern in checkpoint inhibitor combinations [41]. For our current study, a more vigorous monitoring of potential adverse effects was beyond our scope, but previous preclinical reports of concurrent blockage of the immune inhibitory CD96 and PD-1 axes using mAbs at similar dosages revealed a lack of serious immune related or general adverse effects, even after prolonged treatment [17]. Even double knockouts of CD96 and PD-1 in wildtype and MC-38 tumor mice did not significantly compromise immune homeostasis [40]. Moreover, clinical Phase 1 testing of a monoclonal anti-CD96 antibody has recently commenced both as monotherapy and in combination with PD-1 inhibition, highlighting the passing of preclinical safety requirements [42].

In conclusion, interpretations of animal experiments with mAbs have to be taken with care if mAb concentrations were not measured throughout the study. Along with this, negative results of previous in vivo studies with mAbs should be reevaluated if their concentrations were not determined and, if possible, mAb concentrations as well as ADAs should be measured in remaining samples. Our data strongly encourages researchers to reevaluate targets that have been dismissed because of a lack of in vivo effects in proof-of-principle studies using mAbs when their plasma levels have not been determined.

## Data Availability

All pertinent data are included in the manuscript.

## Supplementary Materials

The following supporting information can be downloaded at: https://www.mdpi.com/article/10.3390/biomedicines10092146/s1, Table S1: Mass spectrometry condition. Figure S1: CD96, CD155, and PD-L1 expressions.
