# Supplementary material for: Evaluating Antibody Pharmacokinetics as Prerequisite for Determining True Efficacy as Shown by Dual Targeting of PD-1 and CD96
Source: Biomedicines. 2022 Sep 1;10(9):2146. doi: 10.3390/biomedicines10092146 (PMC9495994; doi:10.3390/biomedicines10092146)
Supplement: Supplementary file 1 [file biomedicines-10-02146-s001.zip › biomedicines-1788205-supplementary.pdf]

Supplementary Files

**LC system**	1290 Inifinity II, Agilent Technologies					
**Analytical column**	Aeris Widepore 3.6µ XB-C8 100x2.10 mm 635557-24 Phenomenex					
**Mobile phase A**	Water 0.1% formic acid					
**Mobile phase B**	Acetonitrile 0.2% formic acid					
**Gradient**	0 min: 95% A, 5% B, 400 µL/min					
	12min: 50% A, 50% B, 400 µL/min					
	13min: 5% A, 95% B, 400 µL/min					
	15min: 95% A, 5% B, 400 µL/min					
**MS/MS system**	ABSciex QTrap 6500+					
**Gas and voltages**	CUR: 40, CAD: 6, ISV: 5000, GS1: 50, GS2: 50, EP: 10, CXP: 11					
	Source temperature: 400°C					
	**Tryptic peptide**	**Transition**	**Retention time [min]**	**Dwell time [ms]**	**DP [V]**	**CE [V]**
anti-CD96 (rat IgG1)	VTSAAFPSPIEK	623.8 → 817.5	3.11	50	77	31
anti-PD1 (rat IgG2a)	VNGSAAFPAPIEK	615.3 → 654.4	2.81	50	76	31
Silumab heavy (IS)	DTLMISR	432.2 → 516.3	3.03	50	62	24

Table S1. LC MS/MS conditions used for the quantitation of anti-CD96 and anti-PD-1. CUR = curtain gas, CAD = collision activated dissociation, ISV = ion source voltage, GS1 = source gas 1, GS2 = source gas 2, EP = entrance potential, CXP = cell exit potential, DP = declustering potential, CE = collision energy.

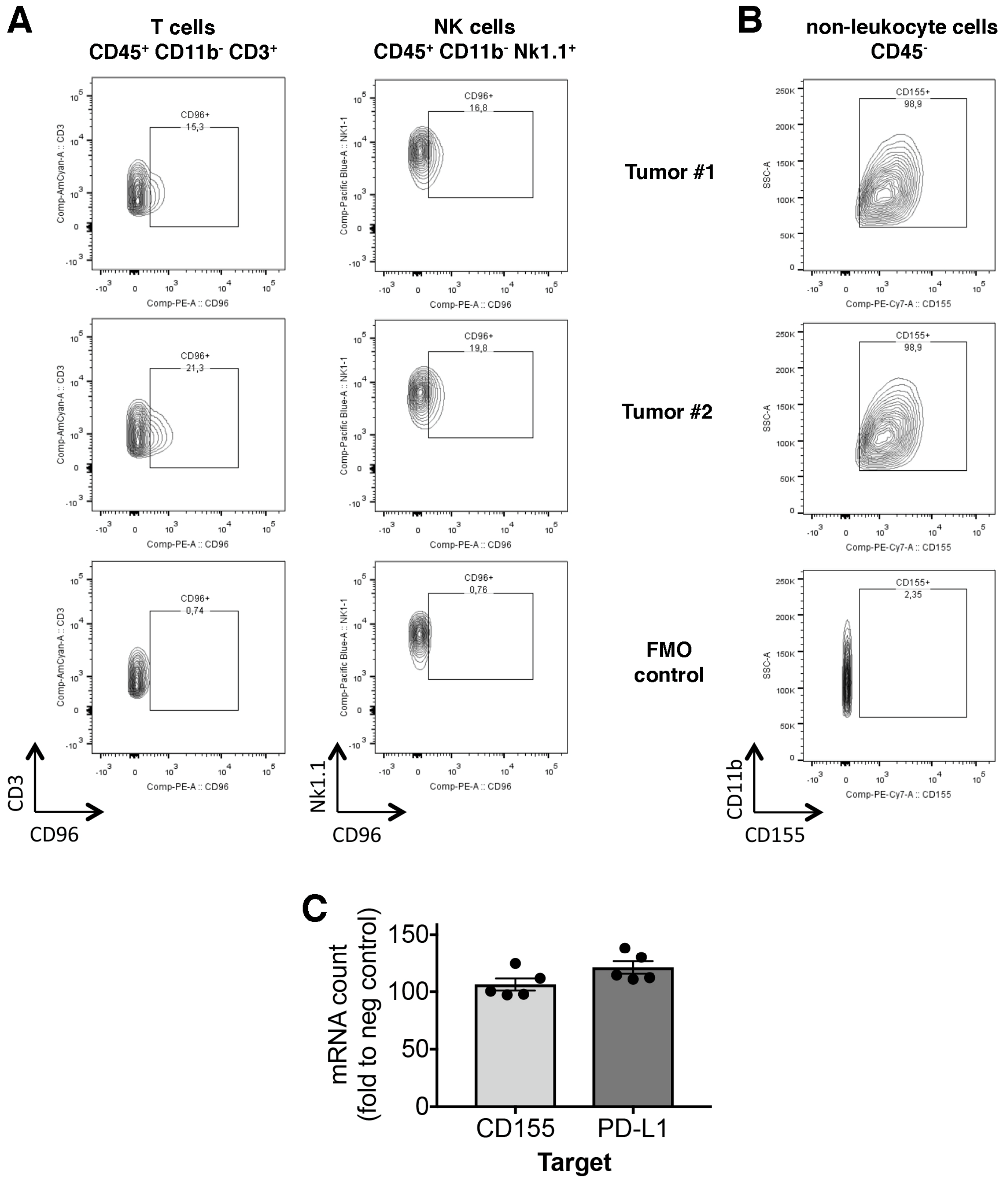

Figure S1. Expression of CD96, CD155, and PD-L1 in MC38 tumors. **A)** CD96 expression on MC38 tumor-infiltrating cells measured by FACS. A subset of tumor-infiltrating T cells (CD45+CD11b-CD3+) as well as NK cells (CD45+CD11b-CD3-NK1.1+) are expressing CD96 in MC38 mice. MC38 tumors from two untreated mice (#1, #2) were excised. FMO: Fluorescence minus one staining control. **B)** CD45 negative cells express CD155, strongly suggesting that tumors and/or stroma express this surface antigen. **C)** Expression of CD96 ligand CD155 and PD-1 ligand PD-L1 in MC38 tumors analyzed using the nCounter Inflammation panel (Nanostring). Tumors were excised 6 d after implantation from 5 isotype-treated mice. RNA expression is presented as normalized counts (negative control = 1). Bar graph is displayed as mean ± SEM (n=5) with symbols reflecting individual tumors.
